# _Remifentanil preconditioning protects against hypoxia-induced senescence and necroptosis in human cardiac myocytes *in vitro*_

**DOI:** 10.18632/aging.103604

**Published:** 2020-06-25

**Authors:** Anna Lewinska, Jagoda Adamczyk-Grochala, Dominika Bloniarz, Beata Horeczy, Slawomir Zurek, Arkadiusz Kurowicki, Bogumila Woloszczuk-Gebicka, Kazimierz Widenka, Maciej Wnuk

**Affiliations:** 1 Department of Biotechnology, Institute of Biology and Biotechnology, University of Rzeszow, Rzeszow, Poland; 2 Anesthesiology and Intensive Care Department with the Center for Acute Poisoning, St. Jadwiga Provincial Clinical Hospital, Rzeszow, Poland; 3 Clinical Department of Cardiac Surgery, St. Jadwiga Provincial Clinical Hospital, Rzeszow, Poland; 4 Medical College, University of Rzeszow, Rzeszow, Poland

**Keywords:** remifentanil, cardiomyocytes, hypoxia, senescence, necroptosis

## Abstract

Remifentanil and other opioids are suggested to be protective against ischemia-reperfusion injury in animal models and coronary artery bypass surgery patients, however the molecular basis of such protection is far from being understood. In the present study, we have used a model of human cardiomyocytes treated with the hypoxia-mimetic agent cobalt chloride to investigate remifentanil preconditioning-based adaptive responses and underlying mechanisms. Hypoxic conditions promoted oxidative and nitrosative stress, p21-mediated cellular senescence and the activation of necroptotic pathway that was accompanied by a 2.2-, 9.6- and 8.2-fold increase in phosphorylation status of mixed lineage kinase domain-like pseudokinase (MLKL) and release of pro-inflammatory cytokine IL-8 and cardiac troponin I, a marker of myocardial damage, respectively. Remifentanil preconditioning was able to lower hypoxia-mediated protein carbonylation and limit MLKL-based signaling and pro-inflammatory response to almost normoxic control levels, and decrease hypoxia-induced pro-senescent activity of about 21% compared to control hypoxic conditions. In summary, we have shown for the first time that remifentanil can protect human cardiomyocytes against hypoxia-induced cellular senescence and necroptosis that may have importance with respect to the use of remifentanil to diminish myocardial ischemia and reperfusion injury in patients undergoing cardiac surgery.

## INTRODUCTION

It is widely accepted that cardiac surgery may result in ischemia and reperfusion of the myocardium leading to perioperative damage, arrhythmia and dysfunction [1]. It has been previously proposed that the heart can tolerate the effects of acute ischemia-reperfusion injury by providing several short cycles of hypoxia and reoxygenation before sustained lethal myocardial ischemia [2]. These cardioprotective effects can be achieved by inducing hypoxia and reoxygenation both before (ischemic preconditioning) and after (ischemic postconditioning) the lethal ischemia [1].

Similar cardioprotective effects to ischemic preconditioning can be observed when using opioids that act *via* opioid receptors, especially the delta opioid receptor (DOR) and the kappa opioid receptor (KOR) that are highly expressed in cardiac tissues [3–5]. Multiple signaling pathways can be implicated in conferring opioid-mediated cardioprotection against ischemia-reperfusion injury, namely reperfusion injury salvage kinases (RISK) pathway involving the activation of Akt and ERK (extracellular signal-regulated kinase) and survivor activating factor enhancement (SAFE) pathway involving the activation of STAT3 (signal transducer and activator of transcription 3) [1, 6]. Clinically used opioids, such as morphine, are usually the mu opioid receptor (MOR) agonists and MOR expression is less abundant in cardiac tissues compared to DOR and KOR expression [1, 7]. Opioid-mediated cardioprotection can be attributed to the weak activity of MOR on the KOR and DOR or receptor crosstalk between the MOR and DOR and in the case of morphine may require the use of high doses that may result in prolonged respiratory depression and sedation limiting its clinical applications [1]. Although, the use of another opioid, namely ultra-short-acting anilidopiperidine opioid remifentanil with adequate protection against intraoperative stimuli and advantageous pharmacokinetics facilitating rapid postoperative recovery might instead be considered [1, 8, 9]. Indeed, remifentanil has been previously reported to promote cardioprotective effects in selected animal models and some clinical settings [10–13]. However, the molecular bases of remifentanil-mediated cardioprotection are complex and far from being understood, and deserve further elucidation especially that remifentanil may confer systemic organ protection against ischemia-reperfusion injury [14, 15].

In the present study, we have considered an *in vitro* model of human cardiomyocytes to investigate the cytoprotection of remifentanil preconditioning against hypoxia-mediated adverse effects. The obtained results are presented and discussed.

## Results and Discussion

### Remifentanil preconditioning attenuates hypoxia-induced senescence in cardiac myocytes

Remifentanil has been found cardioprotective against ischemia-induced injury in a rat model, *in vitro* human myocardium model and on and off pump coronary artery bypass graft (CABG) surgery patients [10–12, 16]. Remifentanil preconditioning approach has been reported to confer more pronounced cardioprotection than other approaches, namely remifentanil postconditioning, ischemic targeting remifentanil, reperfusion targeting remifentanil and both ischemic and reperfusion targeting remifentanil [17]. As the molecular bases of remifentanil-mediated adaptive responses are elusive and far from being understood, in the present study, an *in vitro* cellular model of human cardiac myocytes was used to investigate remifentanil preconditioning-associated effects during hypoxic conditions.

**Figure 1:**
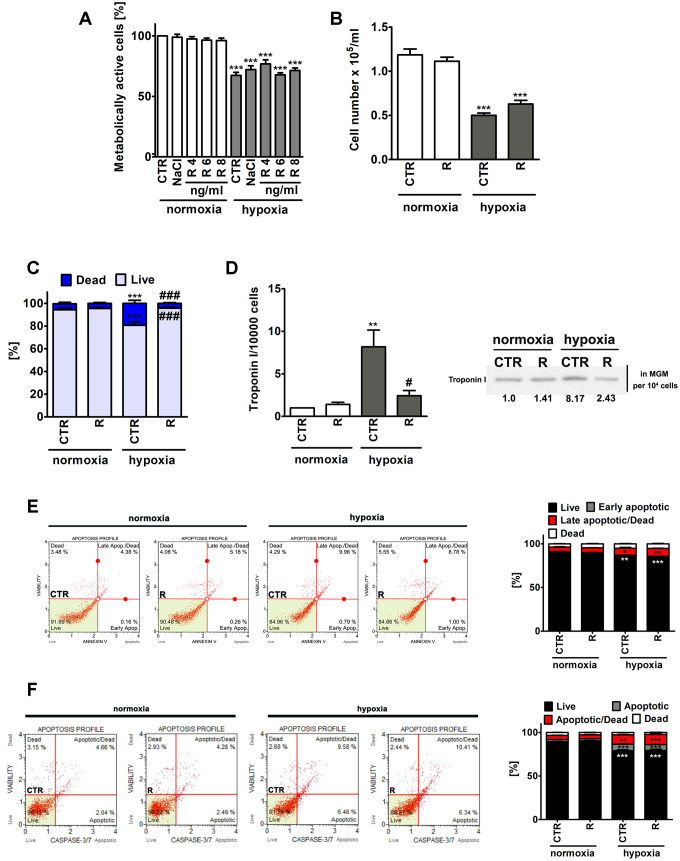
Remifentanil preconditioning-mediated effects on metabolic activity (**A**), cell number (**B**), the levels of necrotic cells (**C**), troponin I release, a marker of myocardial damage (**D**) and the levels of apoptotic cells (**E, F**) during normoxic and hypoxic conditions in human cardiac myocytes (HCM). (**A**) The metabolic activity was assayed using MTT test. Metabolic activity at control normoxic conditions (CTR) is considered as 100%. A solvent action (0.9% NaCl) is also shown. Based on MTT results, the concentration of 8 ng/ml remifentanil was selected for further analysis. Cell number (**B**) and necrotic cell death (**C**) were evaluated using TC10^™^ automated cell counter. (**C**) Necrosis was analyzed using trypan blue exclusion assay. (**D**) Western blot analysis of the levels of cardiac troponin I. Cardiac troponin I levels in supernatants (Myocyte Growth Medium, MGM) were calculated per 10000 cells. Two biomarkers of apoptotic cell death were considered, namely phosphatidylserine externalization (**E**) and the activity of caspase 3/7 (**F**) using Muse^®^ Cell Analyzer and Muse^®^ Annexin V and Dead Cell Assay Kit and Muse^®^ Caspase-3/7 Assay Kit, respectively. Representative dot-plots are also shown. Bars indicate SD, n = 3, ^***^p < 0.001, ^**^p < 0.01, ^*^p < 0.05 compared to normoxic control (CTR), ^###^*p* < 0.001, ^#^*p* < 0.05 compared to hypoxic control (CTR) (ANOVA and Dunnett's *a posteriori* test). CTR, control; R, remifentanil preconditioning.

First, low nanomolar concentrations of remifentanil (remifentanil preconditioning for 24 h, 4, 6 and 8 ng/ml that corresponds to 10.6, 15.9 and 21.25 nM, respectively) were tested both in normoxia and hypoxia (48 h treatment with the hypoxia-mimetic cobalt chloride at 200 μM) and the highest concentration of 8 ng/ml was selected for further analysis on the basis of its relative non-cytotoxicity during hypoxic conditions (Figure 1A). It seems crucial to experimentally adjust an adequate concentration of remifentanil as a high dose of remifentanil has been reported to promote oxidative stress and diminish remifentanil-associated protective effects against ischemia/reperfusion injury in a rat myocardium model [18].

Of course, lowered metabolic activity (MTT assay) and decreased cell number were noticed in control hypoxic conditions compared to control normoxic conditions, however, remifentanil preconditioning did not affect metabolic activity and cell number (Figure 1A and 1B). Remifentanil preconditioning caused a decrease in the number of hypoxia-induced necrotic cells (*p* < 0.001, Figure 1C). Remifentanil preconditioning lowered the number of necrotic cells of about 15% during hypoxic conditions (*p* < 0.001, Figure 1C).

Remifentanil has been previously reported to suppress the release of selected biomarkers of myocardial damage after coronary artery bypass surgery in patients receiving a standardized fentanyl (25 μg/kg) and propofol anesthetic [12]. The addition of remifentanil to the anesthesia regimen (the remifentanil group, n=20, that received a 1 μg/kg bolus followed by a 0.5 μg/kg/min infusion for 30 min after induction but before sternotomy) resulted in decreased levels of creatine kinase (CK-MB), cardiac troponin I (cTnI), ischemia-modified albumin (IMA) and heart-type fatty-acid-binding protein (hFABP) compared to the control group (n=20) that received normal saline [12]. A meta-analysis of 16 randomized controlled trials has also revealed that remifentanil limited cardiac troponin release, the time of mechanical ventilation and the length of hospital stay in patients undergoing cardiac surgery [13]. Thus, we have then decided to investigate if hypoxia may also promote the release of a marker of myocardial damage, namely cardiac troponin I from human cardiac myocytes and the effect of remifentanil preconditioning (Figure 1D). Indeed, elevated levels of cardiac troponin I in the cell culture medium were observed in hypoxic conditions (Figure 1D). A 8-fold increase in the levels of cardiac troponin I was noticed during hypoxia compared to normoxia (*p* < 0.01, Figure 1D) and remifentanil preconditioning reversed this effect (*p* < 0.05, Figure 1D). It has been previously reported that remifentanil preconditioning-based cardioprotection is associated with the delta and kappa opioid receptors (DOR and KOR) and is mediated by the activation of protein kinase C (PKC) and opening of mitochondrial adenosine triphosphate-sensitive potassium channels (mitoK_ATP_ channels) in an isolated rat heart model [19]. However, remifentanil preconditioning also conferred delayed cardioprotection in anaesthetized rats 12-36 h after administration that was mediated by the DOR, KOR and mu opioid receptor (MOR) [20]. More recently, a plethora of signaling transduction pathways, namely phosphatidylinositol 3-kinase (PI3K)/Akt, c-Jun NH_2_-terminal kinase (JNK), extracellular signal regulated kinase (ERK) and Janus activated kinase-2 (JAK2)/signal transducers and activators of transcription 3 (STAT3) pathways have been also implicated in remifentanil preconditioning-mediated cardioprotection against hypoxia/reoxygenation in rat cardiomyocytes and isolated heart [21–23]. Thus, the molecular bases of remifentanil cardioprotective action seem to be multifaceted and require further investigation.

Hypoxia also resulted in apoptotic cell death in human cardiac myocytes as judged by Annexin V staining and caspase 3/7 activity (Figure 1E and 1F). However, apoptosis was much less evident than necrosis (Figure 1). Hypoxia resulted in an increase of necrotic cells of about 14% compared to normoxia (*p* < 0.001, Figure 1C). Phosphatidylserine externalization was increased about 5% and caspase 3/7 activity was increased about 10% in hypoxia compared to control normoxic control (*p* < 0.001, Figure 1E and 1F). Remifentanil preconditioning did not attenuate hypoxia-induced apoptosis (Figure 1E and 1F).

**Figure 2:**
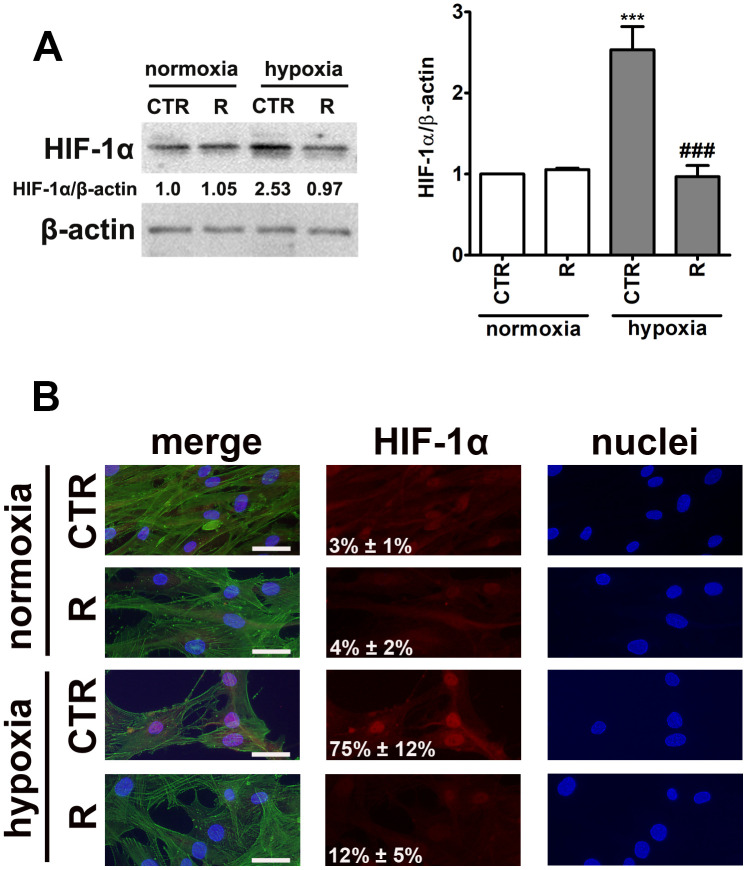
HIF-1α upregulation (**A**) and nuclear translocation (**B**) upon stimulation with hypoxia-mimetic agent cobalt chloride and the effect of remifentanil preconditioning in HCM cells. (**A**) Western blot analysis of the levels of HIF-1α. Data were normalized to β-actin. (**B**) Immunofluorescence analysis of cellular localization of HIF-1α (red). Representative microphotographs are shown, objective 10×, scale bars 10 μm. F-actin staining (green) and nucleus staining (blue) were also considered. Nuclear immuno-signals of HIF-1α were calculated [%]. Bars indicate SD, n = 3, ^***^p < 0.001 compared to normoxic control (CTR), ^###^*p* < 0.001 compared to hypoxic control (CTR) (ANOVA and Dunnett's *a posteriori* test). CTR, control; R, remifentanil preconditioning.

As the hypoxia-mimetic agent cobalt chloride was considered to promote hypoxic-like conditions, we have then analyzed the levels and cellular localization of hypoxia-inducible factor-1 alpha (HIF-1α), a master regulator of oxygen homeostasis that initiates the cellular response to altered levels of oxygen [24] (Figure 2).

Indeed, cobalt chloride treatment resulted in 2.5-fold increase in the levels of HIF-1α compared to control normoxic conditions (*p* < 0.001, Figure 2A). Moreover, cobalt chloride-induced HIF-1α nuclear translocation was observed (Figure 2B). Interestingly, remifentanil preconditioning limited HIF-1α-based response (Figure 2).

**Figure 3:**
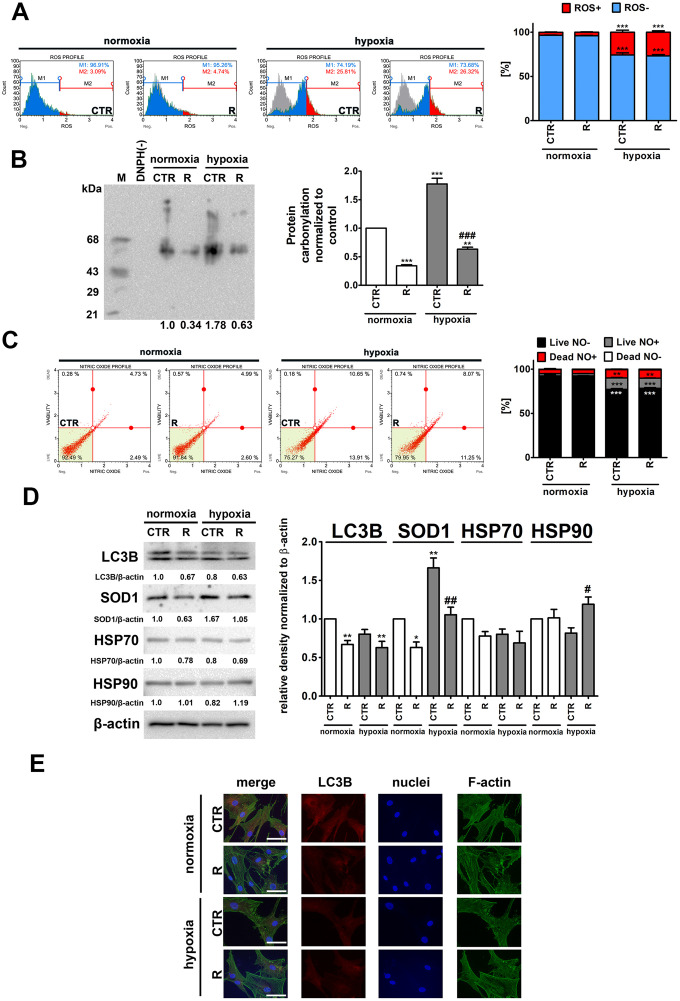
Hypoxia-induced oxidative stress (**A, B**), nitrosative stress (**C**), adaptive oxidative stress, heat shock/chaperone and autophagy-based responses (**D, E**), and the effect of remifentanil preconditioning in HCM cells. (**A**) Superoxide levels were measured using Muse^®^ Cell Analyzer and Muse^®^ Oxidative Stress Kit. Representative histograms are presented. (**B**) Protein carbonylation was investigated using OxyBlot^™^ Protein Oxidation Detection Kit. A negative control without DNPH derivatization (lane DNPH(-)) and a positive control with a mixture of standard proteins with attached DNP residues (lane M) are also shown. The levels of oxidative protein damage were normalized and protein carbonylation during normoxic control conditions was considered as 1. (**C**) Nitric oxide levels were investigated using Muse^®^ Cell Analyzer and Muse^®^ Nitric Oxide Kit. Representative dot-plots are also shown. (**D**) Western blot analysis of the levels of LC3B, SOD1, HSP70 and HSP90. Data were normalized to β-actin. (**E**) Immunofluorescence analysis of cellular localization of LC3B (red). Representative microphotographs are shown, objective 10×, scale bars 15 μm. F-actin staining (green) and nucleus staining (blue) were also considered. Bars indicate SD, n = 3, ^***^p < 0.001, ^**^p < 0.01, ^*^p < 0.05 compared to normoxic control (CTR), ^###^*p* < 0.001, ^##^*p* < 0.01, ^#^*p* < 0.05 compared to hypoxic control (CTR) (ANOVA and Dunnett's *a posteriori* test). CTR, control; R, remifentanil preconditioning.

As hypoxia may promote the production of reactive oxygen species (ROS) and oxidative stress-mediated damage to biomolecules [25], we decided then to evaluate hypoxia-associated oxidative stress, oxidative protein damage (here protein carbonylation) and nitrosative stress and the modulatory effects of remifentanil preconditioning (Figure 3).

As expected, hypoxia resulted in an increase of ROS levels of about 23% and nitric oxide levels of about 11% compared to control normoxic conditions (*p* < 0.001, Figure 3A and 3C). Remifentanil preconditioning did not affect the production of both ROS and nitric oxide (Figure 3A and 3C). In contrast, remifentanil preconditioning protected against hypoxia-induced protein carbonylation (*p* < 0.001, Figure 3B). Hypoxia also promoted the expression of cytosolic superoxide dismutase (SOD1) and the effect was attenuated by remifentanil preconditioning (*p* < 0.01, Figure 3D).

It has been postulated that remifentanil may protect against oxidative stress and hypoxic conditions through the activation of autophagy in different cellular models, namely human fibroblasts, keratinocytes and osteoblasts, and rat cardiomyocytes [26–30]. More recently, it has been reported that remifentanil postconditioning after hypoxia/reoxygenation injury elevated the formation of autophagosomes and promoted autophagosome-lysosome fusion, thereby improving autophagic flux and autophagy promoted cell viability and diminished apoptosis in rat cardiomyocytes [30]. Moreover, the inhibition of autophagy by pharmacological intervention (bafilomycin A1 or chloroquine) and genetic manipulation (ATG7 shRNA) significantly limited remifentanil postconditioning-mediated cardioprotection [30]. In contrast, remifentanil preconditioning did not stimulate autophagy-based adaptive response under hypoxic conditions in human cardiac myocytes (Figure 3D). Remifentanil preconditioning did not increase the levels of LC3II and LC3 puncta (Figure 3D and 3E). Remifentanil preconditioning also did not induce heat shock response (HSR) as judged by the levels of HSP70 (Figure 3D). However, a minor increase in the levels of HSP90 was observed upon remifentanil preconditioning in hypoxic conditions (Figure 3D). This may suggest the involvement of a molecular chaperone HSP90 in the protection against hypoxia-induced oxidative protein damage (Figure 3D).

**Figure 4:**
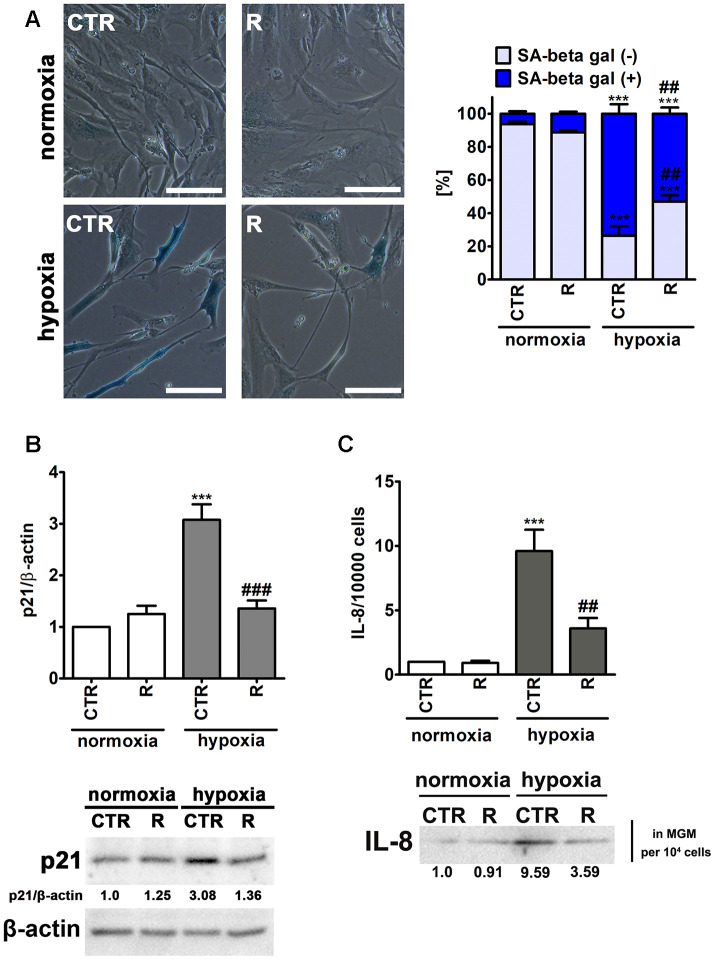
**Remifentanil preconditioning protects against hypoxia-induced senescence in HCM cells.** (**A**) Senescence-associated β-galactosidase (SA-β-gal) activity. Representative microphotographs are shown. Scale bars 50 μm, objective 20×. The levels of SA-β-gal positive (blue) and negative (no blue) cells were calculated [%]. (**B**) Western blot analysis of the levels of cell cycle inhibitor p21. Data were normalized to β-actin. (**C**) Western blot analysis of the levels of pro-inflammatory cytokine IL-8. IL-8 levels in supernatants (Myocyte Growth Medium, MGM) were calculated per 10000 cells. Bars indicate SD, n = 3, ^***^p < 0.001 compared to normoxic control (CTR), ^###^p < 0.001, ^##^p < 0.01 compared to hypoxic control (CTR) (ANOVA and Dunnett's *a posteriori* test). CTR, control; R, remifentanil preconditioning.

As hypoxic conditions promoted oxidative stress and oxidative protein damage (Figure 3) and oxidative stress may be implicated in stress-induced premature senescence (SIPS) [31], we have then asked the question of whether hypoxic conditions may also stimulate cellular senescence in human cardiac myocytes and if remifentanil preconditioning may modulate this phenomenon (Figure 4).

Indeed, hypoxia resulted in increased levels of senescence-associated β-galactosidase (SA-β-gal)-positive cells and p21, a cell cycle inhibitor, and elevated secretion of a pro-inflammatory cytokine IL-8 (Figure 4), all of which are biomarkers of cellular senescence [32]. Hypoxia caused an increase in SA-β-gal-positive cells of about 67% compared to control normoxic conditions (*p* < 0.001, Figure 4A). Moreover, hypoxic conditions promoted 3-fold increase in the expression of p21 and 9.6-fold augmentation of IL-8 secretion compared to normoxia (*p* < 0.001, Figure 4B and C). Interestingly, remifentanil preconditioning diminished pro-senescence effect mediated by hypoxic conditions (Figure 4). Remifentanil preconditioning lowered the levels of SA-β-gal-positive cells of about 21% compared to control hypoxic conditions (*p* < 0.01, Figure 4A) and limited hypoxia-associated elevation in p21 levels and IL-8 secretion (*p* < 0.001, Figure 4B and *p* < 0.01, Figure 4C, respectively). The relationships between hypoxia and cellular senescence are rather complex as both pro- and anti-senescence effects may be observed upon induction of hypoxic conditions [33]. Hypoxia-mediated HIF-1α stabilization may result in HIF-1α binding to *c*-Myc that can induce p21 expression and promote cell cycle arrest [33]. As HIF-1α may stimulate the expression of genes involved in the manifestation of senescence-associated secretory phenotype (SASP), hypoxia can also induce senescence in an autocrine or paracrine fashion [33]. In contrast, anti-senescence effects of hypoxia may be due to HIF-1α-induced expression of glycolytic enzymes, the telomerase subunit (TERT) and HIF-1α-associated negative regulation of p53 activity [33]. Thus, the data on the effects of hypoxic conditions on cell proliferation and induction of cellular senescence are contradictory [34–39]. Hypoxia/reoxygenation limited cell proliferation and induced premature senescence in rat cardiomyocytes that was mediated by elevated levels of p21 [34, 35]. There are no data on remifentanil-associated modulation of hypoxia-induced cellular senescence. However, anti-inflammatory properties of remifentanil may be implicated in remifentanil preconditioning-mediated protection against hypoxia-induced senescence in human cardiac myocytes (this study). It has been reported that remifentanil diminished lipopolysaccharide-associated activation of human neutrophils that was achieved by decreased expression of pro-inflammatory cytokines, namely TNF-α, IL-6 and IL-8 [40]. Remifentanil also decreased the expression of IL-6 in the mouse brain [41]. The authors concluded that remifentanil-mediated anti-inflammatory effect was based on decreased cAMP signaling pathway in lipopolysaccharide-induced neuronal inflammation model [41]. Moreover, a comparison of a continuous infusion of remifentanil with intermittent boluses of fentanyl was considered in the context of perioperative inflammatory activation in coronary artery bypass graft patients under sevoflurane-based anaesthesia [42]. The levels of selected pro-inflammatory parameters, namely TNF-α, IL-6 and IL-8 were significantly higher at some perioperative time points in the fentanyl group compared to the remifentanil group [42]. Remifentanil preconditioning also caused a decrease in the levels of IL-8 in the cell culture medium of human cardiac myocytes that may contribute to observed anti-senescence effect of remifentanil (this study).

### Remifentanil preconditioning abolishes hypoxia-induced necroptosis in cardiac myocytes

**Figure 5:**
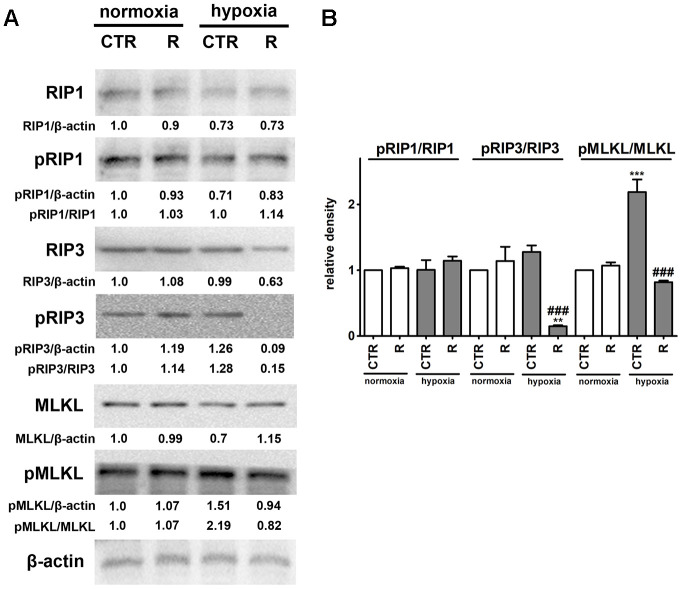
**Remifentanil preconditioning protects against hypoxia-induced necroptosis in HCM cells.** (**A**, **B**) Western blot analysis of the levels of RIP1, phospho-RIP1, RIP3, phospho-RIP3, MLKL and phospho-MLKL. Data were normalized to β-actin. The levels of phospho-RIP1, phospho-RIP3 and phospho-MLKL are also presented as a ratio of phospho-RIP1 to RIP1, phospho-RIP3 to RIP3 and phospho-MLKL to MLKL, respectively. (**B**) Bars indicate SD, n = 3, ^***^p < 0.001, ^**^p < 0.01 compared to normoxic control (CTR), ^###^*p* < 0.001 compared to hypoxic control (CTR) (ANOVA and Dunnett's *a posteriori* test). CTR, control; R, remifentanil preconditioning.

As hypoxia promoted necrotic mode of cell death rather than apoptotic mode of cell death in cardiac myocytes and remifentanil preconditioning protected against hypoxia-induced necrotic cell death (Figure 1), we have then characterized hypoxia-associated necrotic cell death more comprehensively and considered the induction of a regulated form of necrosis, namely necroptosis showing the same morphological features as an accidental necrosis [43–45] (Figure 5).

Necroptosis, a caspase-independent cell death, may be induced by death receptors, interferons, toll-like receptors, intracellular RNA and DNA sensors and others, and mediated by receptor-interacting protein 1 (RIP1, also known as receptor-interacting serine/threonine kinase 1, RIPK1), receptor-interacting protein 3 (RIP3, also known as receptor-interacting serine/threonine kinase 3, RIPK3), and mixed lineage kinase domain-like pseudokinase (MLKL) [44, 45]. More recently, it has been postulated that, in contrast to RIP1, RIP3 and its substrate MLKL are required for necroptotic cell death [44, 45]. Phosphorylation of MLKL by RIP3 results in its conformational changes, insertion and oligomerization in the plasma membrane leading to plasma membrane permeabilization and the release of intracellular content into the extracellular environment, e.g., damage-associated molecular patterns (DAMPs) [44, 45]. Necroptosis can be considered as a trigger of inflammation and can be implicated in some pathophysiological conditions such as viral infection, acute kidney injury and cardiovascular diseases, namely myocardial infarction and cardiac ischemia reperfusion injury [44, 46–48]. Hypoxic conditions resulted in the activation of MLKL (2.2-fold increase in the pools of MLKL phosphorylated form, *p* < 0.001, Figure 5B) that was accompanied by a slight increase in the levels of RIP3 phosphorylated form. However, an increase of about 28% in the phosphorylation status of RIP3 was of no statistical significance (Figure 5B). Hypoxia did not affect the phosphorylation status of RIP1 (Figure 5). Remifentanil preconditioning-mediated diminution in the levels of necrotic cells (Figure 1C) was accompanied by decreased phospho-signals of RIP3 and MLKL (*p* < 0.001, Figure 5B) that may suggest that remifentanil preconditioning may protect against hypoxia-induced necroptosis in human cardiac myocytes (this study). There are no published data on remifentanil-based modulatory effects during hypoxia-induced necroptosis in cellular or animal models. In contrast, hypoxia has been previously recognized as an inducer of necroptotic cell death in different cellular contexts and settings [49–53]. Hypoxia/reoxygenation (H/R) promoted RIP3-dependent mitochondrial fragmentation and necroptotic cell death in H9c2 rat cardiomyocytes [52]. RIP1 or MLKL were not implicated in H/R-mediated necroptosis [52]. In contrast, the activation of dynamin-related protein 1 (Drp1), increased ROS production and decreased mitochondrial membrane potential were observed in necroptotic H9c2 cells [52]. The activation of RIP1 was also not noticed during hypoxia-induced necroptosis in human cardiac myocytes (this study). Necrostatin-1 (Nec-1), an inhibitor of RIP1, protected against hypoxia-induced oxidative stress and necroptosis in mouse skeletal C2C12 myotubes as judged by Nec-1-mediated decrease in the levels of RIP1 and HIF-1α and ROS production [53]. Oxidative stress was also involved in hypoxia-induced necroptosis in mouse hepatocytes as a suppressing effect was observed upon treatments with antioxidants trolox and *N*-acetyl-cysteine (NAC) [51]. Perhaps hypoxia-induced oxidative stress may also promote both cellular senescence and MLKL-mediated necroptosis in human cardiac myocytes (this study). MLKL silencing using siRNA technology also attenuated necroptotic cell death in primary rat cortical neurons subjected to oxygen-glucose deprivation and caspase inhibitor zVAD treatment (a model of neuronal necroptosis and ischemic brain injury) [49]. The pharmacological inhibition (GSK’872) and genetic silencing (siRNA) of RIP3 also suppressed hypoxia-mediated necroptotic cell death and ischemic brain injury using *in vitro* and *in vivo* mouse models, namely HT22 hippocampal neurons and C57BL/6 mice that was accompanied by decreased levels of HIF-1α [50]. Remifentanil preconditioning also resulted in diminished HIF-1α signaling upon cobalt chloride treatment (this study).

**Figure 6:**
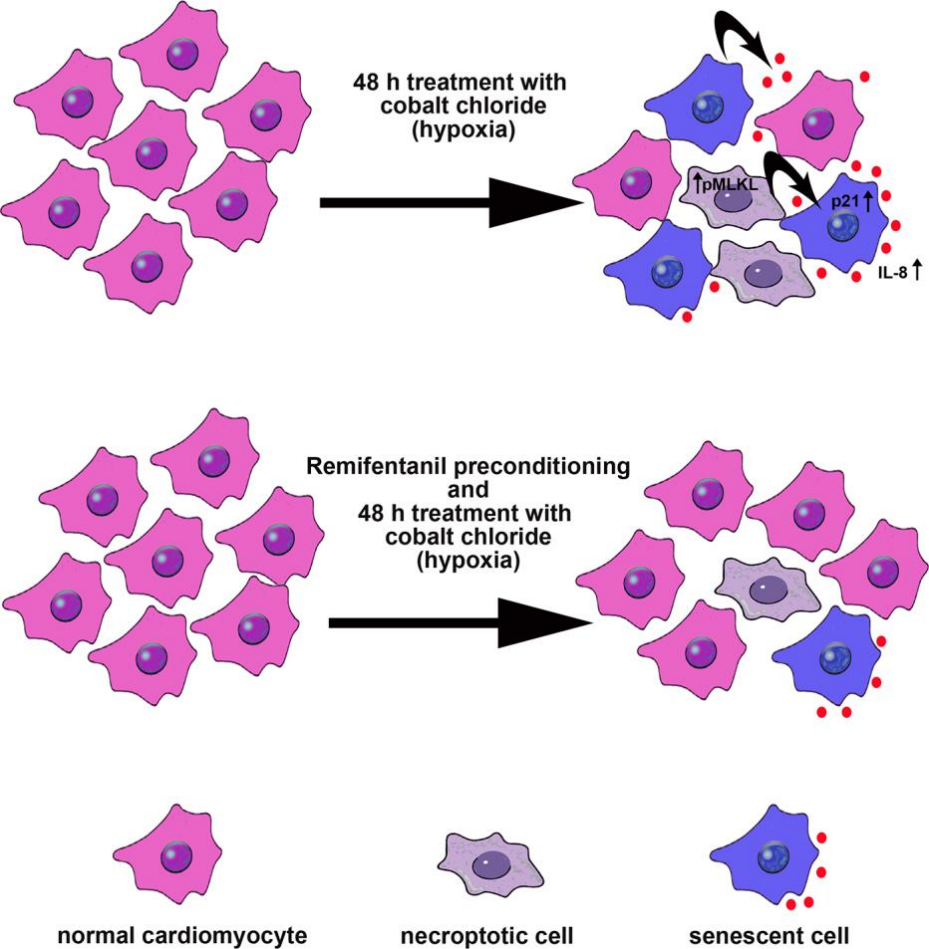
**Remifentanil preconditioning protects against hypoxia-induced cellular senescence and necroptosis in human cardiac myocytes that is achieved by remifentanil preconditioning-mediated decrease in the levels of cell cycle inhibitor p21, secretion of IL-8 proinflammatory cytokine as a part of senescence-associated secretory phenotype (SASP) and phospho-MLKL-based necroptotic signaling.** Cellular senescence and necroptosis may be due to hypoxia-mediated oxidative stress (not shown). Moreover, necroptotic cells may release some proinflammatory signals (e.g., IL-8) that may also promote cellular senescence and then senescent cells may further stimulate the occurrence of other senescent cells by IL-8 signaling (black arrows). All these adverse effects can be reversed by remifentanil preconditioning.

In conclusion, we have shown for the first time that remifentanil preconditioning may also protect against hypoxia-induced cellular senescence and MLKL-mediated necroptotic cell death in human cardiac myocytes that, at least in part, may be achieved by remifentanil preconditioning-associated decrease in oxidative protein damage, p21 levels, IL-8-based proinflammatory signaling and MLKL activity (Figure 6).

## MATERIALS AND METHODS

### Cell culture and remifentanil preconditioning

Primary human cardiac myocytes (HCM, lot 436Z024.4, catalog number PC-C-12810, two independent vials) isolated from the ventricles of the adult heart (33-year-old Caucasian female) were obtained from PromoCell GmbH (Heidelberg, Germany) and cultured at 37°C in a low-serum Myocyte Growth Medium (MGM) containing 5% fetal calf serum (FCS), recombinant human epidermal growth factor (0.5 ng/ml), recombinant human basic fibroblast growth factor (2 ng/ml), recombinant human insulin (5 μg/ml) (PromoCell GmbH, Heidelberg, Germany), 100 U/ml penicillin, 0.1 mg/ml streptomycin and 0.25 μg/ml amphotericin B (Corning, Tewksbury, MA, USA) in a cell culture incubator in the presence of 5% CO_2_. HCM cells were passaged using Trypsin/EDTA ratio of 0.04%/0.03% designed for gentle detachment of adherent primary human cells according to the manufacturer’s instructions (PromoCell GmbH, Heidelberg, Germany). Cells were seeded at the concentration of 10000 cells per cm^2^ of a 25 cm^2^ culture flask or a 6-well plate, cultured overnight, pre-treated with 8 ng/ml remifentanil hydrochloride (Ultiva, lot U22B, dissolved in sterile 0.9% NaCl) (Aspen Pharma Ireland Limited, Dublin, Ireland) for 1 h and then cultured for 48 h in the presence of 200 μM cobalt chloride (Merck KGaA, Darmstadt, Germany).

### Selection of remifentanil concentration

A non-cytotoxic concentration of remifentanil (8 ng/ml) was selected on the basis of MTT test. HCM cells were seeded onto a 96-well plate at the concentration of 5000 cells per a well and after 24 hours of culture, remifentanil was added at the concentrations ranging from 4 to 8 ng/ml for 1 h and then cells were cultured for 48 h in the presence of 200 μM cobalt chloride. Metabolic activity (MTT assay) was then assayed as previously described [54]. Metabolic activity at normoxic conditions was considered as 100%.

### Cell number

The cell number was automatically calculated using TC10^™^ Automated Cell Counter (Bio-Rad, Hercules, CA, USA).

### Apoptotic and necrotic mode of cell death

Apoptosis was evaluated using Muse^®^ Cell Analyzer and Muse^®^ Annexin V and Dead Cell Assay Kit and Muse^®^ Caspase-3/7 Assay Kit. Briefly, to analyze phosphatidylserine externalization, Annexin V staining [54] was used and to investigate the activity of caspase 3/7, a DEVD peptide substrate conjugated to a DNA-binding dye was used. Caspase 3/7-mediated cleavage of DEVD peptide substrate resulted in the release of the dye, its translocation to the nucleus and binding of the dye to DNA and high fluorescence. A marker of cell death, 7-aminoactinomycin D (7-AAD) was also considered. 7-AAD cannot be excluded from late apoptotic/dead cells. Thus, four subpopulations of cells can be revealed, namely live cells (Annexin V negative, 7-AAD negative or caspase 3/7 activity negative, 7-AAD negative), early apoptotic cells (Annexin V positive, 7-AAD negative or caspase 3/7 activity positive, 7-AAD negative), late apoptotic cells/dead cells (Annexin V positive, 7-AAD positive or caspase 3/7 activity positive, 7-AAD positive) and dead (necrotic) cells (Annexin V negative, 7-AAD positive or caspase 3/7 activity negative, 7-AAD positive). Moreover, necrosis was also investigated using trypan blue dye exclusion assay. Briefly, cells were incubated with 0.4% trypan blue and then dead cells with porous cell membranes (blue-stained cells) were automatically scored using TC10^™^ Automated Cell Counter (Bio-Rad, Hercules, CA, USA).

### Oxidative and nitrosative stress

Superoxide levels were analyzed using Muse^®^ Cell Analyzer and Muse^®^ Oxidative Stress Kit and protein carbonylation was evaluated using an OxyBlot^™^ protein oxidation detection kit (Merck KGaA, Darmstadt, Germany) as previously described [55]. Nitric oxide levels were investigated using Muse^®^ Cell Analyzer and Muse^®^ Nitric Oxide Kit according to manufacturer’s instructions (Merck KGaA, Darmstadt, Germany).

### Senescence-associated β-galactosidase activity (SA-β-gal)

HCM cells were pre-treated with 8 ng/ml remifentanil for 1 h and then cultured for 48 h in the presence of 200 μM cobalt chloride. After 7 days of cobalt chloride removal, SA-β-gal activity was assayed as comprehensively described elsewhere [56].

### Immunofluorescence

Immunostaining protocol was used as comprehensively described elsewhere [54]. Briefly, fixed cells were incubated with primary antibodies anti-HIF-1α (ab51608, 1:500) and anti-LC3B (ab52862, 1:500) (Abcam, Cambridge, UK) at 4°C overnight and secondary antibody conjugated to Texas Red (1:1000, T2767) (Thermo Fisher Scientific, Waltham, MA, USA) at room temperature for 1 h. Nuclei were visualized using DAPI staining and F-actin was detected using phalloidin staining. Digital cell images were captured using an Olympus BX61 fluorescence microscope equipped with a DP72 CCD camera and Olympus CellF software.

### Western blotting

Protein extraction and Western blotting protocol was used as previously described [57]. The following primary and secondary antibodies were used: anti-HSP70 (PA5-14521, 1:500), anti-HSP90 (MA1-10373, 1:1000), anti-RIP1 (3493T, 1:500), anti-phospho-RIP1 (Ser166) (65746T, 1:500), anti-RIP3 (13526T, 1:1000), anti-phospho-RIP3 (Ser227) (93654T, 1:1000), anti-MLKL (14993T, 1:500), anti-phospho-MLKL (Ser358) (91689T, 1:500), anti-HIF-1α (ab51608, 1:1000), anti-p21 (PA5-14949, 1:1000), anti-LC3B (ab52862, 1:1500), anti-SOD1 (PA5-23245, 1:2500), anti-IL-8 (ab154390, 1:250), anti-troponin I (ab52862, 1:750) and HRP-linked antibodies anti-β-actin (A3854, 1:40000), anti-mouse IgG (7076, 1:3000) and anti-rabbit IgG (7074, 1:3000) (Thermo Fisher Scientific, Waltham, MA, USA, Merck KGaA, Darmstadt, Germany, Abcam, Cambridge, UK and Cell Signaling Technology, Danvers, MA, USA). The data represent the relative density normalized to β-actin. Phospho-RIP1, phospho-RIP3 and phospho-MLKL signals were also normalized to RIP1, RIP3 and MLKL signals, respectively. The levels of IL-8 and troponin I in post-culture supernatants (spent MGM) were calculated per 10^4^ cells as previously reported [58].

### Statistical analysis

The results represent the mean ± SD from at least three independent experiments. The obtained data conform the assumptions of the analysis of variance (ANOVA) as evaluated using Shapiro-Wilk normality test and Levene test for the equality of variances. Statistical significance of differences between remifentanil preconditioning and normoxic control as well as remifentanil preconditioning and hypoxic control was evaluated using one-way analysis of variance (ANOVA) with post-hoc testing using a Dunnett’s multiple comparison test. *P*-values of less than 0.05 were considered significant. Statistical analysis of the data was performed using a GraphPad Prism 5.
